# Emerging role of human microbiome in cancer development and response to therapy: special focus on intestinal microflora

**DOI:** 10.1186/s12967-022-03492-7

**Published:** 2022-07-06

**Authors:** Hourieh Sadrekarimi, Zhanna R. Gardanova, Morteza Bakhshesh, Farnoosh Ebrahimzadeh, Amirhossein Fakhre Yaseri, Lakshmi Thangavelu, Zahra Hasanpoor, Firoozeh Abolhasani Zadeh, Mohammad Saeed Kahrizi

**Affiliations:** 1 Department of Immunology, Faculty of Medicine, Tbriz University of Medical Science, Tabriz, Iran; 2 Department of Psychotherapy, Pirogov Russian National Research Medical University (Pirogov Medical University), 1 Ostrovityanova St., 117997 Moscow, Russia; 3 Molecular and Medicine Research Center, Khomein University of Medical Sciences, Khomein, Iran; 4 Department of Internal Medicine, Faculty of Medicine, Mashhad University of Medical Sciences, Mashhad, Iran; 5 Faculty of Medicine, Qazvin University of Medical Sciences, Qazvin, Iran; 6 Department of Pharmacology, Saveetha Dental College, Saveetha Institute of Medical and Technical Science, Saveetha University, Chennai, India; 7 Department of Immunology, Faculty of Medical Sciences, Tarbiat Modares University, Tehran, Iran; 8 Department of Surgery, Faculty of Medicine, Kerman University of Medical Sciences, Kerman, Iran; 9 Alborz University of Medical Sciences, Karaj, Alborz Iran

**Keywords:** Microbiome, Dysbiosis, Cancer development, Bacterial manipulation

## Abstract

In recent years, there has been a greater emphasis on the impact of microbial populations inhabiting the gastrointestinal tract on human health and disease. According to the involvement of microbiota in modulating physiological processes (such as immune system development, vitamins synthesis, pathogen displacement, and nutrient uptake), any alteration in its composition and diversity (i.e., dysbiosis) has been linked to a variety of pathologies, including cancer. In this bidirectional relationship, colonization with various bacterial species is correlated with a reduced or elevated risk of certain cancers. Notably, the gut microflora could potentially play a direct or indirect role in tumor initiation and progression by inducing chronic inflammation and producing toxins and metabolites. Therefore, identifying the bacterial species involved and their mechanism of action could be beneficial in preventing the onset of tumors or controlling their advancement. Likewise, the microbial community affects anti-cancer approaches’ therapeutic potential and adverse effects (such as immunotherapy and chemotherapy). Hence, their efficiency should be evaluated in the context of the microbiome, underlining the importance of personalized medicine. In this review, we summarized the evidence revealing the microbiota's involvement in cancer and its mechanism. We also delineated how microbiota could predict colon carcinoma development or response to current treatments to improve clinical outcomes.

## Introduction

The human gut microbiome is the genomic content of the gut microbiota, which comprises all microorganisms that are colonized in the human gastrointestinal tract (GIT), such as viruses, fungi, protozoa, and predominantly bacteria [1]. The diversity and composition of normal gut microbiota were altered throughout the individual life span and shaped by factors such as dietary nutrients, mode of delivery, age, geographical area, use of antibiotics, and host genetics [2–5]. They serve specific functions in producing short-chain fatty acids (SCFAs), immune system homeostasis, nutrient and drug metabolism, vitamin synthesis, and protection against pathogen colonization [6–8].

Progress in sequencing technologies and bioinformatics tools have facilitated large-scale microbiome studies, such as the Human Microbiome Project (HMP) and the MetaHIT (Metagenomics of the Human Intestinal Tract) funded by the U.S. National Institutes of Health (NIH) and the European Commission, respectively to understand the impact of the microbiome on human health and disease [9–12]. Gut dysbiosis is defined as the functional and compositional alterations of gut microbiota in response to environmental or host-related changes associated with the manifestation, detection, or therapy of the disease [13]. It is increasingly appreciated that gut dysbiosis relates to various conditions such as inflammatory bowel disease (IBD), neurodegenerative disease [e.g., Parkinson’s and Alzheimer’s disease(A.D.)], diabetes mellitus (D.M.), and cancer [14–18]. Moreover, altered gut inhabitant microbes influenced the development of autoimmune diseases, i.e., rheumatoid arthritis, spondyloarthritis, and systemic lupus erythematosus (SLE) [19–21].

Cancer is defined as a group of diseases characterized by uncontrolled cell proliferation and caused by genetic and environmental factors [22, 23]. A growing body of research has revealed the link between microbiota and cancer, particularly stomach cancer. For instance, the variation in the gastric microbial composition in the various stages of carcinogenesis including superficial gastritis, atrophic gastritis, gastric intraepithelial neoplasia and gastric cancer, was reported by Zhang et al. [24]. Alteration in gut flora increases the risk of gastrointestinal malignancy and potentiates carcinogenesis by inducing chronic inflammation, producing mutagenic metabolites, modifying stem cell dynamics, and stimulating cell proliferation [25]. In addition, the imbalanced microbial community could be affected the genetic and epigenetic mechanisms of colorectal cancer development [26]. Epigenetic alteration regulates gene expression through the changes in miRNA regulation, DNA methylation, and histone modification. Hence, gut microbiome involvement in carcinogenesis could be mediated by deregulating the epigenetic modifications [27, 28]. For instance, induction of malignant phenotype in murine gastric tissue by *Helicobacter pylori* is associated with hypomethylation and downregulation of miR-490-3p [29]. Accordingly, cancer-related microbes were associated with specific miRNA expression and other epigenetic modifications that regulate genes involved in cancer-related pathways.

The diversity and composition of gastrointestinal microbes are impacted not only by cancer development but also influence the anti-tumor immunity and response to therapy [30]. Hence, multiple strategies have been recommended to manipulate microbiota in cancer treatment, e.g., probiotics and fecal microbiota transplantation (FMT) [30, 31].

In addition to bacterial components, the microbiome comprises fungal communities known as the mycobiome, which influence cancer development [32]. As documented by Luan et al. the fungal diversity in adenoma was lower compared with adjacent biopsy samples. Moreover, operational taxonomic units (OTUs) revealed substantial differences between adenomas and adjacent tissues, as well as between advanced and non-advanced samples. For instance, *Fusarium* and *Trichoderma* were enriched in the adjacent biopsy samples of advanced and non-advanced adenoma, respectively [33]. According to Coker et al. a higher ratio of Basidiomycota/Ascomycota and alteration in enteric fungal diversity in patients with colorectal cancer (CRC) is associated with the colorectal carcinogenesis [34]. Furthermore, the fungal diversity was higher in late-stage CRC than in early-stage CRC [35]. It’s worth noting that we’re focusing on the bacterium's role in cancer development.

Elucidating the interaction between microbiota and various stages of cancer development (i.e., initiation, promotion, and progression) might shed light on the gut flora’s specific function in cancer prevention or treatment. This review intends to delineate the role of the microbiome in cancer development and therapy and recapitulate the strategies that manipulated the microbiota to improve cancer treatment (see Table 1).

**Table 1:** Association between bacterial colonization and cancer development

Bacteria	Types of tumor	References
*Salmonella* *typhi*	↑ Gallbladder cancer	Di Domenico et al. [212]
*Helicobacter* *pylori*	↑ Gastric cancer	Wang et al. [213]
Uropathogenic *Escherichia* *coli*	↑ Prostate cancer	Elkahwaji et al. [214]
*Escherichia* *coli* (strain CP1)	↑ Prostate cancer	Simons et al. [215]
*Escherichia* *coli*	↑ Bladder cancer	El-Mosalamy et al. [216]
*Bacteroides* *vulgatus*, *Bacteroides* *stercoris*	↑ Colorectal cancer	Hu et al. [79]
*Lactobacillus* *acidophilus*, Lactobacillus S06, and *Eubacterium* *aerofaciens*	↓ Colorectal cancer	Hu et al. [79]
Fusobacteria, Leptotrichia genus	↓ Pancreatic cancer	Fan et al. [217]
*Porphyromonas* *gingivalis*, *Aggregatibacter* *actinomycetemcomitans*	↑ Pancreatic cancer	Fan et al. [217]
↑↑↑ Enterotoxigenic *Bacteroides* *fragilis*	Colorectal cancer	Haghi et al. [218] Zamani et al. [219]
↑↑↑ *Fusobacterium* *nucleatum*	Colorectal cancer	Chen et al. [220]
↑↑↑ *Porphyromonas* *gingivalis*, *Fusobacterium* *nucleatum*	Oral squamous cell carcinoma	Chang et al. [221]
↑↑↑ Enterobacteriaceae	Stomach cancer	Youssef et al. [222]
↓↓↓ Bifidobacteriaceae	Rectal neoplasm	Youssef et al. [222]
↑↑↑ Capnocytophaga, Veillonella (in saliva)	Lung cancer	Yan et al. [223]

Some bacterial species diminished or raised the risk of various types of cancer as shown by the direction of the arrows, i.e., ↓ or ↑

On the other hand, the comparison of the microbial composition between patients and healthy individuals revealed the higher/lower abundance of the bacterial population as indicated by ↑↑↑and ↓↓↓, respectively

## Association between microbiome and cancer development

Accumulating evidence sheds light on the association of microbiota with cancer initiation and progression, including the studies conducted on germ-free and gnotobiotic animals (i.e., animals born and raised under aseptic conditions and colonized with specific microorganisms) and their comparison with their conventional counterparts, which presented convincing evidence for microbiota's involvement in tumor induction and the establishment of local and systemic immune responses [36, 37]. In some of these investigations, researchers found a higher frequency of liver cancer, a lower incidence of small intestinal polyposis, and little or no difference in the occurrence of mammary tumors in germ-free mice [38–40]. Moreover, the incidence of tumors induced by carcinogens in germ-free (GF) and conventional (CV) animals exhibited contradictory results, which could be related to differences in animal strains, tumor induction protocol (including type, dose, and route of administration of carcinogen), and organs involved in carcinogenesis [37, 41–45].

The oral cavity is inhabited by various types of the microbial community that could be translocated to the other sites of the body, as well as tumor tissue, and involved in cancer initiation and progression by multiple pathways (such as producing inflammatory mediators, inhibiting immune response, and inducing malignant transformation) [46]. It has been confirmed that some oral bacterial species could have reached the intestinal flora and contributed to gut dysbiosis [47]. This imbalanced gut microbiota may have been attributed to gastrointestinal diseases and cancer. The association between oral microbiota and tumor development is documented by several reports [48, 49]. For instance, the genomic analysis of colorectal adenocarcinoma tissue revealed the *F. nucleatum* and other *Fusobacterium* species enrichment [50]. In addition, these data were consistent with a meta-analysis conducted by Drewes et al. that elucidated increased levels of oral microbiota such as *Peptostreptococcus stomatis*, *Parvimonas micra*, and *Fusobacterium nucleatum* on CRC tissue [51]. Accordingly, it has been suggested that the analysis of oral microbiota could be considered as a noninvasive cancer biomarker [52, 53].

Sears et al. proposed a paradigm for describing the role of microbiota in the causation of colorectal cancer, in which three models were considered: individual microbes (model 1), a microbiota population (model 2), and individual microbes that interact with the microbial community (model 3) [54]. The causal role of individual microbes in carcinogenesis could be determined by reproducibly generating specific cancers in mice [55]. For instance, the carcinogenic potential of enterotoxigenic Bacteroides fragilis (ETBF) was evaluated in multiple intestinal neoplasia (Min) mouse strain. Additionally, the rate of carcinogenesis after antibiotic therapy could be determined in order to assess the collaboration and synergistic effect of other microbiota members [56]. Considering the first model, individual microbes (such as *Streptococcus gallolyticus*, *Enterococcus faecalis*, *Enterotoxigenic Bacteroides fragilis*, *Escherichia coli*, and *Fusobacterium nucleatum*) may contribute to colorectal cancer pathogenesis by triggering inflammation and DNA damage or impairing the DNA repair [54]. Contrarily, as an example of illustrating the contribution of the microbial community, the study conducted by Wong et al. provided evidence to confirm the tumorigenesis effect of altered gut microbiota in CRC patients. Increased infiltration of T-helper (Th) 1 and Th17 cells, upregulation of genes involved in the pro-inflammatory response (including interleukin (IL) 17A, IL-22, IL-23A, CXC chemokine receptor (CXCR) 1, and CXCR2), and the oncogenic pathway were observed in intestinal tissue of germ-free and conventional mice gavaged by feces from CRC patients [57]. The bacteria associated with specific human cancers are listed below (Table 2).

**Table 2:** Oncogenic potential of bacterial toxin

Bacterium	Toxin	Oncogenic activity
*Helicobacter* *pylori*	Cytotoxin-associated gene A (CagA)	Binding and activating the SHP-2 tyrosine phosphatase [224, 225] Disrupting the polarity of epithelial cells [103, 226] Increasing cell survival by cyclin D1 induction [227] Cell scattering by binding to Grb2 [228] Stimulating MMP10 expression [229] Inhibiting apoptosis by downregulating Siva1 protein [230] Enhancing cell proliferation by upregulating reg3 [231]
*Helicobacter* *pylori*	Vacuolating toxin (VacA)	Vacuolation of gastric epithelial cells [232, 233] Disrupting the integrity of epithelial cells leading to carcinogen penetration [234] Inhibiting acid secretion from gastric parietal cells that proper the stomach microenvironment for the colonization of other bacterial species [235, 236] Interfering with protective immunity by suppressing the function of immune cells [237, 238] Causing cell death via necrosis and apoptosis [239–241] Stimulating pro-inflammatory activity [238, 242]
*Pasteurella* *multocida*	Pasteurella multocida toxin (PMT)	Eliciting mitogenic effect through Gq-signaling pathways [243, 244] Preventing apoptosis by inducing pim1 and Akt pathway and modulating the expression of Bcl2 family [245] Promoting the activation of STAT transcription factor and other signaling pathways involved in carcinogenesis [246]
*Escherichia* *coli*	Cytotoxic necrotizing factor 1 (CNF1)	Impacting several cellular processes (e.g., inflammation, survival, cell adhesion, and motility) by modifying Rho GTPases activity and actin cytoskeleton arrangement [247–249] Activating the RhoC in bladder cancer cells leading to HIF-1α expression, VEGF secretion, and promoting angiogenesis [250] Promoting the migration and invasion of prostate cancer cells [251] Inducing epithelial-mesenchymal transition[252]
*Escherichia* *coli*	Colibactin	Inducing cell proliferation [253, 254] Diminishing tumor-infiltrating lymphocytes (CD3^+^ T population) [255]
Enterotoxigenic *Bacteroides* *fragilis*	Bacteroides fragilis enterotoxin (BFT)	Enhnaced cleavages the E-cadherin that leads to disruption of intercellular junction, release and nuclear translocation of β-catenin, c-myc upregulation, and cell proliferation [256, 257] Increasing mucosal permeability [258] Inducing oncogenic inflammation by activating NF-κB [259–261]

*SHP-2* SH2 domain-containing protein tyrosine phosphatase-2 (SHP-2), *Grb2* Growth factor receptor-bound protein 2, *MMP10* Matrix metalloproteinase 10, *Siva1* Apoptosis-Inducing Factor, *Reg3* Regeneration gene 3, *Pim1* Proviral integration site for Moloney murine leukemia virus-1, *Akt* protein kinase B, *BCL2* B-cell lymphoma 2, *STAT* Signal transducer and activator of transcription, *VEGF* vascular endothelial growth factor, *RhoC* Ras homolog family member C, *HIF1α* hypoxia-inducible factor 1α, *SENP1* SUMO1/sentrin specific peptidase 1, *CDK1* cyclin-dependent kinase 1

It should be noted that some bacteria cause cancer, while others hasten tumor growth by suppressing the immune system and promoting cancer cell proliferation. As an example for the latter groups, *F. nucleatum* colonized malignant tissue selectively through the interaction between fibroblast activation protein 2 (Fap2) lectin and upregulated galactose/N-acetyl-galactosamine (Gal–GalNAc) on tumor cells, which accelerated tumor growth and metastasis [58, 59]. Rubinstein and colleagues proposed a two-hit model for colorectal cancer development. The first hit is provided by accumulated mutation, and the second hit is delivered by microbes (i.e., *F. nucleatum*) that accelerate tumor growth [60]. By this fact, the early identification of carcinogenic bacteria colonization would be beneficial for cancer treatment and prevention.

The impact of the intestinal flora on tumor development can be categorized as direct or indirect mechanisms [61]. For instance, *Fusobacterium nucleatum* directly contributes to colorectal tumorigenesis by binding Fusobacterium adhesin A (FadA) to E-cadherin, which leads to β-catenin activation and cellular proliferation [62, 63]. On the contrary, persistent stimulation of the immune system is one of the indirect mechanisms whereby microbiota affects carcinogenesis.

## Microbial mechanisms of carcinogenesis and tumor progression

### Oncogenic actions of microbiota through the induction of chronic inflammation

Inflammation is a double-edged sword that can be anti- and pro-tumorigenic [64]. The inflammatory mediators (cytokines and reactive oxygen and nitrogen species) produced elevated mutation rates and DNA damage in the tumor microenvironment. They reduced the expression and activity of DNA repair systems, resulting in the genetic instability of cancer cells. Indeed, the inflammatory microenvironment impacts diverse aspects of carcinogenesis, like tumor cell transformation, proliferation, invasion, metastasis, and angiogenesis, and could be regarded as the seventh hallmark of cancer [65, 66]. Microorganisms contribute to tumor initiation and progression by inducing tumor-promoting inflammation or translocating to the tumor site and persisting cancer-induced inflammation [67, 68]. Studies such as those conducted by Wei et al. have elucidated that inflammation is a crucial component between microbiota and patient survival or prognosis of colorectal cancer. The higher frequency of some microbial species in the worse prognosis group, such as *Fusobacterium nucleatum,* was correlated with the upregulation of TNF-α (tumor necrosis factor), β-catenin, and NF-κB (nuclear factor-kappa B). It also induces a shift ftom pro-inflammatory M1-phenotype to a tumor-promoting M2-phenotype. Conversely, *Faecalibacterium prausnitzii* was elevated in the survival group and was associated with less expression of NF-κB, β-catenin, and MMP9 (matrix metallopeptidase 9) [69].

The different components of the immune system, particularly innate immunity, are essential in the relationship between inflammation induced by commensal microbiota and carcinogenesis. The recognition of pathogen-associated molecular patterns (PAMPs) of commensal microflora by toll-like receptors (TLRs) plays a crucial role in maintaining intestinal homeostasis. At the same time, their dysregulated interaction may cause chronic inflammation [70]. According to research conducted by Fukata and coworkers, the TLR4 was upregulated in colon cancer samples from patients with chronic ulcerative colitis and animal model of colitis-associated cancer (CAC) and contributed to the colon carcinogenesis by activating the EGFR (epidermal growth factor receptor) signaling [71]. Moreover, the studies on the spontaneous intestinal tumor model confirmed the critical role of MyD88 (adaptor protein triggered by TLRs) in tumor development [72]. Related to this, the production of toxic products by the gut microbiota reduces the integrity of the mucosal surface that allowing foreign antigens to penetrate more easily and generating local inflammation [73]. It is pertinent to point out that the persistent activation of NF-kB transcription factors in response to chronic inflammation and dysregulation of the Wnt/β-catenin signaling pathway contributes to tumor development [74–76].

**Fig. 1:**
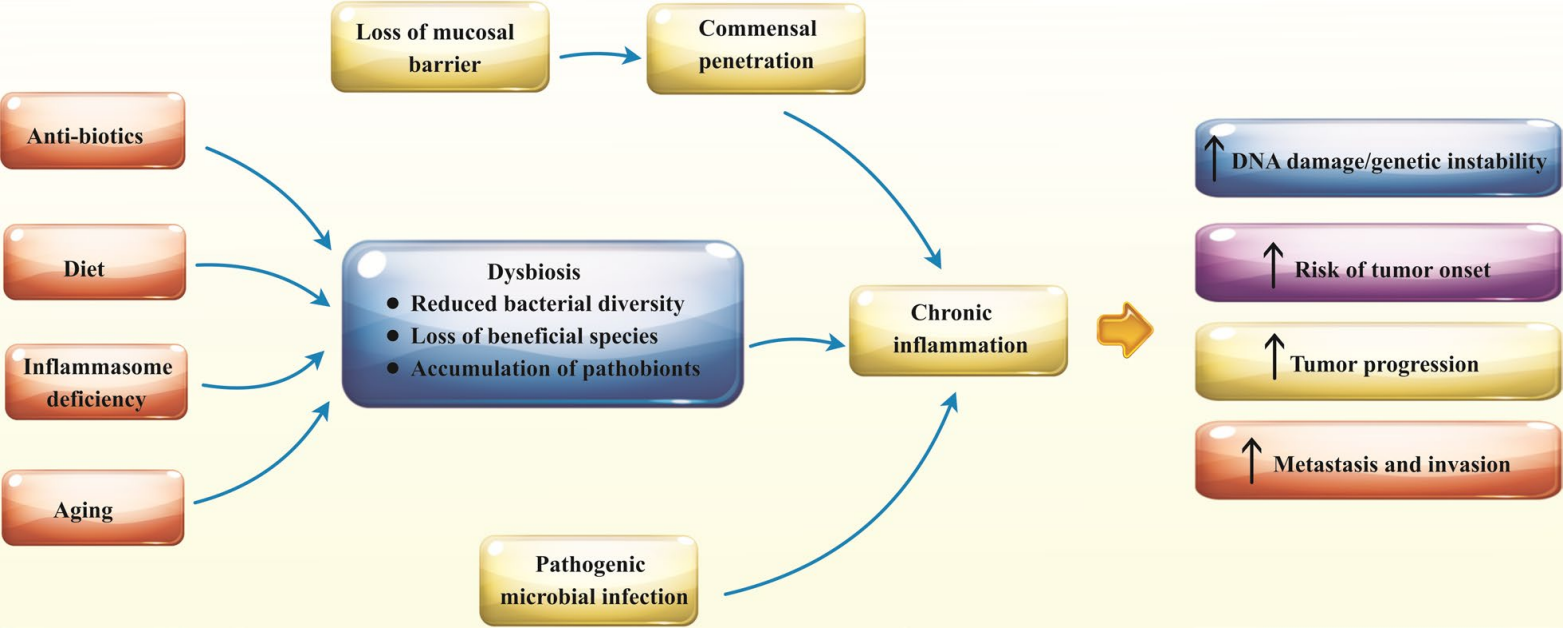
Gut microbiota contribution in cancer development through inflammation induction

The inflammasome is a cytosolic multiprotein complex consisting of NOD-like receptors (NLRs), adaptor protein ASC (Apoptosis-associated speck-like protein containing a CARD), and pro-caspase-1. Following activation of the inflammatory pathway by recognition of pathogen-associated molecular patterns (PAMPs) or damage-associated molecular patterns (DAMPs) with the relevant receptors, inflammasome assembles and cleaves pro-caspase-1, which in turn cleaves pro-forms of the cytokines, such as pro-IL-1β and pro-IL-18, and converts them to the bioactive forms [77]. Functional activity of this complex protein is required for modulating the colonic microbial population, and deficits in any part of these constituents lead to the inflammation caused by altered gut microbiota [78]. Using the Azoxymethane (AOM)/Dextran Sodium Sulfate (DSS) model, Hu et al. illustrated that altered microbiota of Inflammasome deficient mice causes inflammation-induced colorectal cancer through the induction of CCL5 (CC-chemokine ligand 5) and activation of the IL-6 signaling pathway in intestinal epithelial cells [79, 80]. Likewise, based on the determinative role of NOD2 (Nucleotide-binding oligomerization domain-containing protein 2) in the regulation of intestinal microbiota composition, gut dysbiosis following NOD2 deficiency augmented the risk of colitis and colitis-associated colorectal cancer in mice [81–83]. Interestingly, the carcinogenic phenotype associated with NOD2-mediated microbiota dysbiosis was transferable and could be transferred via fecal microbiota transplantation from NOD2 deficient mice to wild-type (WT) or germ-free mice with NOD2 sufficient expression [81]. As represented in Fig. 1, several factors are modified the microbial equilibrium leading to tumor induction and progression by generating inflammation.

In addition to the pivotal role of innate immunity in modulating gut microbiota composition to regulate inflammation and cancer development, members of the adaptive immune response, such as T-helper 17 and regulatory T cells, are also implicated in tumorigenesis. Disturbance of the microbiota balance (dysbiosis) and an increase in the abundance of Th17-inducing bacteria can cause chronic inflammation that leads to the onset and progression of cancer. Studies confirmed that tumoral inflammation driven by Th17 cells is often tumor-promoting and linked to a poor prognosis in colorectal cancer [84, 85].

Activating the IL-23 signaling pathway after Toll-like receptors sense translocated microbes or microbial products, and triggering the MyD88 adaptor activation, is critical for producing downstream cytokines, i.e., IL-17 [84]. Furthermore, the production of ATP by commensal microbiota has been proposed as the mediator for naturally occurring and/or pathogenic Th17 cell differentiation in lamina propria. As documented by Atarashi et al. the bacterially-driven ATP activates CD70^high^ CD11c^low^ cells to produce IL-6, IL-23, and TGF-β (Transforming growth factor beta), which subsequently promote Th17 differentiation [86].

Notably, multiple investigations have pointed to Colonization with the specific bacterial species that promote Th17 differentiation. For instance, *segmented filamentous bacteria* (SFB) produced serum amyloid A (SAA), which triggers Th17 cells differentiation by acting on dendritic cells in the lamina propria [87]. This Th17 response stimulated by some members of commensal microbiota contributes to infection-induced carcinogenesis. Colonization with enterotoxigenic *Bacteroides fragilis*, for illustration, promotes IL-17-producing cells-driven inflammation and the development of colon cancer [88, 89].

It is now well established that cyclophosphamide (CTX) treatment altered the composition of intestinal microflora and stimulated the gram-positive bacteria translocation to secondary lymphoid organs, leading to naive T cell polarization towards Th1 and Th17 cells [90, 91]. Interestingly, the CTX-stimulated pathogenic Th17 response mediated by dysbiotic microbiota is tumor suppressive and prevents the outgrowth of cancer cells. It’s worthwhile to point out that numerous studies documented the contradictory role of Th17 cells in cancer development [92–94]. These cells have a pro-tumorigenic effect by inducing angiogenesis and genetic instability and activating the IL-6-oncogenic STAT-3 signaling pathway. On the other hand, they are involved in anti-tumor immunity by converting to Th1 cells (and producing IFN-) and eradicating tumor cells directly or promoting tumor-specific immune cell recruitment [93]. This dual function could be due to Th17 cells' plasticity and heterogeneity [95]. In addition, the type and stage of the tumor are also effective.

On the other hand, Regulatory T cells attenuate inflammation-induced carcinogenesis in an IL-10-dependent manner. In this regard, the adoptive transfer of regulatory T cells reduced the onset and progress of inflammatory bowel disease (IBD) and colon cancer induced by *Helicobacter hepaticus* colonization in aged Rag2 (Recombination activating gene 2 protein)-deficient mice [96, 97]. Similarly, the polysaccharide A (PSA) of nontoxigenic *Bacteroides fragilis* reduced IL-17 production and protected from *H. hepaticus*-driven colitis by inducing IL-10 production in CD4 T cells [98].

### Carcinogenic action of microbiota through the production of toxins and metabolites

While the importance of the microbiome in carcinogenesis has been proven, further research is required to elucidate the precise mechanism whereby the microbial components contribute to cancer development and treatment. Aside from inflammation-driven by microbiota, which acts as an indirect mechanism for carcinogenesis, some bacterial metabolites directly contribute to the causation of cancer [99]. Bacterial toxins are one of these components that can interact with several signaling pathways that modulate cancer-related biological processes, including proliferation, cell cycle, differentiation, and apoptosis [100]. Table 2 lists some bacterial species that generated toxins with oncogenic potential. For instance, the cytotoxin-associated gene A (CagA) of *Helicobacter pylori*, a bacterial oncoprotein linked to the development of gastric cancer by promoting the genetic instability and interactions with host cell proteins like SHP2 (Src homology two phosphatases), E-cadherin, and PAR1 (Partitioning-defective 1) [101–104]. Some consequences of this interaction include inhibition of apoptosis, increased survival and cell proliferation, loss of cellular polarity, and neoplastic transformation [105].

As another example, the enterotoxigenic strains of *Bacteroides fragilis* (ETBF) are a risk factor for colorectal cancer, and its metalloprotease toxin is implicated in the process of triggering mucosal inflammation [106–108]. According to Goodwin et al. *B. fragilis* enterotoxin (BFT) promotes spermine oxidase enzyme (SMO) upregulation in intestinal epithelial cells, which leads to the production of reactive oxygen species (ROS) and DNA damage [109]. Additionally, BFT exposure causes morphologic changes, E-cadherin cleavage, and cell proliferation stimulation, which is at least in part mediated by E-cadherin cleavage followed by β-catenin nuclear translocation and upregulation of the proto-oncogene *c-myc* [110–112].

The production of metabolites may also contribute to the oncogenic action of gut microbiota. Undigested food components are breakdown by the microbial community into several metabolites, some of which have been shown to have a protective and or harmful effect on cancer development (Table 3) [113]. Short-chain fatty acids (butyrate, propionate, and acetate) are one of the most critical products of carbohydrate fermentation, with anti-inflammatory, anti-proliferative, and apoptotic inducing effects [113, 114]. It also assists in maintaining intestinal homeostasis by elevating Foxp3 IL-10-producing Treg cells [115, 116]. Similarly, Lactobacillus acidophilus, Lactobacillus, linoleic acids (CLAs), Lactobacillus casei, Lactobacillus bulgaricus Lactobacillus plantarum, Bifidobacterium infantis, Bifidobacterium breve, Bifidobacterium longum, and Streptococcus thermophiles exhibited anti-neoplastic and pro-apoptotic impacts [117, 118]. It should be mentioned that some evidence, such as that published by Nakashima et al. has shown a link between GPR40 (G protein-coupled receptor 40) expression, as CLAs receptor, and colorectal cancer progression and worse prognosis [119, 120].

**Table 3:** Metabolites with cancer prevention effects

Dietary components	Metabolites	Microbes	Anti-cancer effect	References
Fiber	Short-chain fatty acids (such as butyrate, propionate, and acetate)	For instance: *Holdemanella biformis, Faecalibaculum rodentium, Clostridium butyricum*	Anti-inflammatory, anti-proliferative, apoptotic inducing effects, elevating Foxp3 IL-10-producing Treg cells	Smith et al. [115] Li et al. [262] Furusawa et al. [116] Louis et al. [113]
Linoleic acids	Conjugated linoleic acids (CLAs)	*Lactobacillus acidophilus*, *Lactobacillus casei*, *Lactobacillus bulgaricus*, *Lactobacillus plantarum*, *Bifidobacterium infantis*, *Bifidobacterium breve*, *Bifidobacterium longum*, and *Streptococcus thermophiles*	Anti-neoplastic, pro-apoptotic	Maggiora et al. [118] Ewaschuk et al. [117]
Polyphenols (such as: phenolic acid, flavonoids, lignin, anthocyanin,)	Low-molecular-weight phenolic acids	For instance: *Escherichia coli, Eubacterium sp., Lactobacillus sp., Bifidobacterium sp., Bacteroides sp.*	Chemoprevention effect, reducing cell prolifration, increasing apoptosis, anti-inflammatory effect, modulating enzymes	Bultman [263] Cardona et al. [264]

On the other side, microbial fermentation of a high-fat or high-protein diet generates NOCs (N-nitroso compounds), polyamines, ammonia, hydrogen sulfide, and secondary bile acids, which promote tumor development by triggering inflammation and DNA damage [113]. As reviewed by Bernstein and coworkers, short-term exposure of cells to bile acids resulted in the production of reactive oxygen and nitrogen species (ROS/RNS), which subsequently enhanced DNA damage, mutation rates, and apoptosis. Over a more extended period, mutant cells acquired growth advantages like apoptosis resistance, raising the risk of gastrointestinal tract cancer [121]. Moreover, secondary bile acid (especially deoxycholic acid) induces cancer cell proliferation and invasion by activating the β-catenin signaling pathway [122].

Overall, microbiome involvement in cancer initiation and progression can be mediated by targeting the tumor cells directly or indirectly by modulating the immune system. In this context, microbes can influence different components of innate and adaptive immunity (including dendritic cells, natural killer cells, myeloid cells, CD8 T cells, etc.) and cancer progression via direct (act as an antigen) and indirect mechanisms (by producing byproducts and cytokines). In the former, homology between microbial epitopes and tumor antigens induced cross-reactive T cells that can contribute to anti-tumor immunity [123]. As discussed in multiple studies, the molecular mimicry and presence of cross-reactive T CD8 and T CD4 cells may improve the efficacy of anti-cancer approaches [124, 125]. Besides, recognition of bacterial antigens by pattern recognition receptors (PRRs) (like TLRs and NLRs) leads to activation of downstream signaling cascades (NF-κB and STAT3 activation) and production of pro-and anti-inflammatory cytokines. PAMPs activated Dendritic cells (DCs) and other antigen-presenting cells leading to traveling to mesenteric lymph nodes where they activate T helper cells. Notably, microbial dysbiosis and over-activation of NF-κB, STAT3, and Wnt/β-catenin signaling pathways are contributed to cancer pathogenesis by regulating anti-tumor immune response, promoting inflammation, and inducing cancer cell proliferation and metastasis [126].

### Other factors involved in intestinal cancer progression

In a bidirectional relationship, cancer cells and microbes can potentiate each other. For instance, the colibactin-producing *Escherichia coli* (*E. coli*) is more prevalent in colon tissue of patients with colorectal cancer than in diverticulosis patients [127]. The genotoxic compound colibactin promoted cellular senescence and growth factor production that subsequently stimulated cell proliferation and tumor growth [128, 129]. Moreover, increasing evidence indicates the bacterial involvement in metastasis progression of colorectal cancer. Cancer cells' detachment from original sites and their traveling to the new organ or tissue is called metastasis cascade. According to Seely et al. specific bacterial species like *E. coli* influences multiple steps of this process, including dissemination, circulation, colonization, and proliferation through the epithelial-mesenchymal transition (EMT), biofilm formation, paired migration, and altering the local microenvironment [130].

The production of flavonoids, anti-oxidants, SCFAs, and vitamins following carbohydrate-rich diet consumption increased the intestinal barrier integrity, reduced DNA damage and inflammation, and limited pathogen colonization. On the contrary, high fat or high protein diets that metabolized to carcinogenic products (such as N-nitroso compounds and secondary bile acids) enhanced cancer progression by promoting DNA damage, tumor proliferation, and inflammation [131]. It’s pertinent to point out that excess energy uptake and obesity increase cancer risk by altering microbial composition and metabolism.

## Cancer-preventing properties of microbes

Unlike cancer-promoting microbiota, which are contributed to cancer development by inducing inflammation or producing carcinogenic compounds, some microbial species have beneficial effects on cancer prevention. In this context, probiotics effectively control gastrointestinal inflammatory disorders like inflammatory bowel disease, which have been associated with an increased risk of colorectal cancer [132]. Multiple in vitro and in vivo studies demonstrated various aspects of probiotics in cancer onset and progression, including their effects on cell proliferation reduction, apoptosis induction, and cell cycle arrest. Probiotics’ cancer-prevention effects could be attributed to a variety of mechanisms, including (1) maintaining colon homeostasis, for example, by pH regulation; (2) modulating intestinal microflora composition and their metabolic activity; (3) binding and inactivating carcinogens; (4) producing anti-carcinogenic metabolites such as SCFAs and conjugated linoleic acid; and (5) immunomodulatory effects like phagocytes activation, which results in the early eradication of cancer cells [133, 134]. *Lactobacillus* and *Bifidobacterium* are the two most common probiotics in the digestive system. *Lactobacillus* prevents cancer by producing antioxidants and anti-angiogenesis factors, reducing inflammation and DNA damage, and preventing polyamines and tumor-specific antigens expression [135].

## Impacts of microbiota on anti-tumor immunity and therapy

Tumor development and intestinal microbiota impact each other in a reciprocal relationship. Any alteration in the composition of the gut microbes influences the tumor microbiota and the tumor microenvironment, thus affecting cancer progression. Given the dual role of the immune system in dampening or promoting cancer, the crosstalk between microbiota and tumor could be mediated by the immunomodulatory activity of microflora. It has been verified that gut flora or its metabolites (such as SCFAs) affects multiple aspects of host immune response.

Commensal microbes have a role in the development and maturation of the host immune system, so any alteration in the microbial community caused by antibiotic usage, diet, and other environmental factors could influence the cancer immune surveillance [136]. For instance, *Fusobacterium nucleatum* inhibited the tumor-killing activity of natural killer (NK) cells, and the higher level of it inversely correlated with a lower density of CD3 T cells in colorectal carcinoma tissue [137, 138]. Moreover, disrupting the gut microbiota and reducing intestinal SCFAs by using broad-spectrum antibiotics promoted macrophage hyper-activation and Th1 pro-inflammatory response [139]. Due to the study documented by Ma et al. modulating commensal microbiota controlled hepatic natural killer T (NKT) cell accumulation and liver tumor growth by modifying bile acids metabolism [140]. The number of monocytes/macrophages and conventional DCs in the ileum and spleens increased in gnotobiotic pigs after colonization with two strains of lactic acid bacteria [141]. Furthermore, peritoneal macrophages of germ-free mice have higher lysosomal activities and reduced C3b-receptor-mediated phagocytosis than control, which reached the normal level following cohousing with conventional mice [142]. Similarly, as reported by Ohkubo and coworkers, germ-free rats are neutropenic and have altered functions [143, 144]. Commensal microflora is involved in TCD4 + differentiation, so any alteration in their composition imbalanced the T helper and biased the immune response toward the specific subtypes [145]. For instance, some species of commensal bacteria like *Bacteroides fragilis* and *segmented filamentous bacteria* induce anti-inflammatory or pro-inflammatory responses through promoting T cell differentiation toward Treg or Th17 cells, respectively [146].

In addition, gut flora may influence systemic immunity and tumor development by producing metabolites (such as short-chain fatty acid and secondary bile acids), whole bacterial translocation, stimulating cytokine secretion, circulation of primed lymphocytes, and antigen cross-reactivity [30]. Although microbiota in all barriers contributes to the local immune response, the gut microflora is mainly responsible for the microbiota's systemic influence [147]. Also, due to the specific features of tumor tissue like hypoxia, abundant nutrients, immune evasion strategies served by cancer cells, and neoangiogenesis, the tumor microenvironment can be a supportive and immune-protected site for bacterial colonization, as proposed by Heymann et al. [148]. These intra-tumoral bacteria could influence the tumor infiltrated immune cells. For instance, *Fusobacterium nucleatum* within tumors reduces the anti-tumor activity of NK cells and other immune cells via its Fap2 protein interaction with TIGIT (T-cell immunoglobulin and ITIM domain) inhibitory receptors [138].

Parallel to the oncogenic action of microbiota, multiple studies pointed to its possible role in modulating the effectiveness of chemotherapy and immunotherapy approaches [149–154]. As reviewed by Alexander and colleagues, the modulatory mechanisms of gut microbiota on the efficacy and toxicity of chemotherapy treatment can be categorized as follows: translocation, metabolism, diversity reduction, enzymatic degradation, and immunomodulation [150]. It means that chemotherapy agents altered the composition of gut microbiota and disrupted the integrity of the intestinal barrier that facilitated commensal bacteria translocation to the systemic milieu. Cyclophosphamide, for instance, alters the intestinal microbiota and promotes the translocation of gram-positive bacteria to secondary lymphoid organs, where they mediate Th1 and Th17 cells generation [90, 151]. Moreover, gut flora regulates the immune and inflammatory response induced by chemotherapy and also modifies pharmaceutical, leading to thepotentiating or attenuating the drug efficacy or enhacing its adverse effects [150, 152]. Using the mouse model, Iida and colleagues proved that microbiota affects the pro-inflammatory response required for oxaliplatin treatment [153].

Accordingly, there is a bidirectional relationship between the gut microbiota and anti-cancer treatment (especially chemotherapy). It means that changes in the gut microbiota affect the efficacy and toxicity of chemotherapy medications and that chemotherapy, in turn, can alter the microbial composition. In comparison to healthy controls, the overall number and diversity of microbial communities were reduced during chemotherapy treatment in patients with acute myeloid leukemia. This change is linked to a decrease in the number of anaerobic bacteria, which lowers pathogen colonization resistance and increases the frequency of pathogenic enterococci [155]. Similarly, cyclophosphamide administration enhanced pathogenic species like *Pseudomonas*, *E. coli*, enterococci, and *Enterobacteriaceae* [156]. Chemotherapy-induced gut microbiota dysbiosis has been associated with the colonization of pathogenic bacteria, intestinal damage, and the development of adverse effects in several other studies. Despite extensive research on the impact of chemotherapy on the microbiota, there are fewer reports of dysbiosis derived from other anti-cancer medications [31, 157]. In the study documented by Vetizou et al. treatment of ipilimumab [anti-CTLA-4 (Cytotoxic T-lymphocyte antigen 4)] was associated with elevated *Clostridiales* abundance and a decrease in *Burkholderiales* and *Bacteroidales* [125]. In addition, comparing the microflora of CRC patients before and after surgery revealed that total bacterial counts and the counts of some obligate anaerobes (including *Clostridium coccoides*, *Bacteroides fragilis*, *Clostridium leptum*, *Atopobium*, *Bifidobacterium*, and *Prevotella*) were reduced, whereas *Pseudomonas*, *Enterobacteriaceae*, *Staphylococcus*, and *Enterococcus* increased [158].

Immunotherapy strategies (e.g., immune checkpoint inhibition by anti–programmed death-1 (PD-1)/anti–PD-1 ligand 1 (PD-L1) and anti-CTLA-4 therapy, and adoptive cell transfer) could restore the impaired immune response against cancer cells, while their efficacy influenced by any factors that affect immune surveillance, such as the microbial community of the gastrointestinal tract [159–161]. Besides, these interventions showed interpatient heterogeneity that may rely on genetic and environmental factors. Several studies highlighted the crucial role of gut microbiota in clinical response and or primary resistance to immunotherapy approaches. According to Sivan et al. the difference in the gut microbiota composition, particularly *Bifidobacterium*, influences anti-cancer immunity and the therapeutic efficacy of anti-PDL1 therapy by altering DC maturation and tumor-specific T cell response [162]. Fecal microbiota transplantation from immune checkpoint inhibitors (ICIs) responding and non-responding patients to germ-free or prescribed antibiotics mice confirmed the gut microbiota attribution in primary resistance to anti-PD1 blocked [163]. Analysis of patients’ fecal samples elucidated that *Akkermansia muciniphila* was correlated with clinical response to ICIs and enriched in ICIs responder patients [163]. As reported by Mager and colleagues, the ICB (Immune checkpoint blockade) therapies-promoting effects of specific bacterial species, including *Bifidobacterium pseudolongum*, *Akkermansia muciniphila*, and *Lactobacillus johnsonii*, are mediated by secretion and systemic translocation of inosine and hypoxanthine that affect T cell differentiation and activity via adenosine A2A receptor (A_2A_R) in a context-dependent manner [164].

Tanoue et al. proposed 11 strains that augmented the frequency of intestinal IFN-γ CD8 T cells and potentiated the response to immune checkpoint inhibitory therapy in the MC38 murine colon adenocarcinoma model [165]. Similarly, in melanoma patients undergoing anti-PD1 immunotherapy, the fecal microbiota analysis revealed higher diversity and *Faecalibacterium* enrichment in responder patients [166]. Tumor-infiltrated and systemic immune response assessments in patients enriched in the *Faecalibacterium* in the gut microbiome (*Ruminococcaceae* family, *Clostridiales* order) indicated TCD8 accumulation in tumor tissue and the higher frequency of effector T lymphocytes in the systemic circulation. Contrarily, Individuals with enrichment of *Bacteroidales* displayed a higher frequency of Treg and myeloid-derived suppressor cells (MDSCs) in their circulation [166]. Several other studies reported an association between gut microbiota and the effectiveness of anti-cancer approaches in various malignancies (Table 4). Results could be due to discrepancies in how patients were classified as responders or non-responders and differences in the analysis method [167].

**Table 4:** Association between commensal microbiome composition and clinical outcomes of immunotherapy

Therapy	Types of malignancy	Responder	Non-responder	References
Anti-PD1	NSCLC	*Akkermansia muciniphila, Enterococcus hirae*		Routy et al. [163]
Anti-PD1	Melanoma	*Faecalibacterium*	*Bacteroidales*	Gopalakrishnan et al. [166]
Anti-PD1	Metastatic melanoma	*Bifidobacterium longum, Collinsella aerofaciens*, and *Enterococcus faecium*	*Ruminococcus obeum, Roseburia intestinalis*	Matson et al. [167]
Anti-CTLA4	Metastatic melanoma	*Firmicutes phylum*, e.g. *Faecalibacterium*	–	Chaput et al. [201]
Anti-CTLA4 Anti-PD1	Metastatic melanoma	*Bacteroides caccae*, *Streptococcus parasanguinis*	–	Frankel et al. [265]
Anti-CTLA4 Anti-PD1	Metastatic melanoma	*Faecalibacterium prausnitzii*, *Holdemania filiformis, Bacteroides thetaiotamicron*	–	Frankel et al. [265]
Pembrolizumab	Metastatic melanoma	*Dorea formicigenerans*	–	Frankel et al. [265]

*Anti-PD1* anti-programmed cell death 1 protein, *Anti-CTLA-4* anti- cytotoxic T-lymphocyte-associated protein 4

It is pertinent to point out that microbiota may reduce the responsiveness to immune checkpoint blockade; thus, microbial transplantation or TNF-α blockade could be beneficial in circumventing the resistance [168]. The abundances of various species of gut flora regulated the efficacy of these approaches through the secretion of pro-inflammatory cytokines (e.g., IL-12 and IFN-γ), elevating regulatory cells (e.g., MDSC and Treg), increasing/decreasing the maturation or function of DC, and enhancing the frequency of effector cells in circulation or tumor sites (e.g., cancer-specific TCD8 + and TCD4 +) [169].

## Manipulating the microbiota for cancer therapy

Given the dual role of the bacteria in suppressing or supporting tumorigenesis, manipulating the bacterial community would be beneficial in cancer prevention and treatment. In this regard, multiple strategies have been proposed to alter gut microflora composition: (1) Oral administration of certain types of bacteria classified as probiotics, (2) Using prebiotics (non-digestible food components that are well fermented by beneficial bacteria but not pathogens) to stimulate the growth and activity of beneficial intestinal bacteria, (3) Applying the combination of probiotics microorganisms with prebiotic components to reap the benefits of both strategies (known as synbiotics), (4) Targeting cancer-associated bacteria with antibiotics and (5) Fecal microbiota transplantation (FMT) [157, 170, 171].

With respect to other approaches (applying probiotics and prebiotic components), fecal microbiota transplantation is worked quicker and is more effective in the reconstitution of the intestinal microflora [172]. Moreover, FMT showed more success in controlling *Clostridium difficile* infection because feces contain additional metabolites such as proteins, bacteriophages, and bile acids [173].

The gut microbiota composition affects both the effectiveness of anti-cancer therapies and the adverse effects (such as chemotherapy, radiotherapy, and immunotherapy). Thus, combining anti-cancer treatments with antibiotics or commercially available probiotic supplements, which provide beneficial bacterial species, may help to improve clinical efficacy and manage side effects [174]. Irinotecan (CPT-11) is a camptothecin derivative that acts as an anti-neoplastic medication by inhibiting topoisomerase I. The glucuronide hydrolysis from SN-38G (the inactive form of SN-38 detoxified in the liver) in the intestinal lumen by bacterial β-glucuronidase causes dose-limiting diarrhea [175]. Studies applied diverse tactics to reduce the incidence and severity of Irinotecan-induced-diarrhea, including administration of antibiotics or probiotics and selective inhibition of β-glucuronidase [175–177]. Similarly, surveys such as that conducted by Chitapanarux and coworkers have shown that receiving live *lactobacillus acidophilus* with *Bifidobacterium bifidum* during pelvic radiotherapy lowers the severity and incidence of diarrhea [178]. Likewise, oral administration of *Bifidobacterium* probiotics attenuated colitis following Ipilimumab (anti-CTLA-4) without impairing its therapeutic efficiency in tumor control [179].

Given that *F. nucleatum*-colonized colorectal cancer suppressed the anti-cancer activity of infiltrated-T and NK cells and augmented cancer cell proliferation, tumor growth, and metastasis, targeting this anaerobe gram-negative bacteria (e.g., with antibiotics) could be valuable for *Fusobacterium*-enriched CRC treatment [180–182]. It should be noted that the consumption of antibiotics may cause unwanted and non-specific depletion of bacterial species [157]. To reduce the undesirable effects of conventional antibiotics, it’s better to apply pathogen-specific antibiotics with a narrow range and preferential cytotoxicity for bacterial species [183, 184].

As another example, an elevated abundance of *Bacteroides fragilis* following anti-CTLA4 treatment in melanoma patients boosted the clinical response to ipilimumab [125]. Hence, oral administration of *B. fragilis*, transfer of T cells reactive to *B. fragilis*, and immunizing with polysaccharides of *B. fragilis* were all employed to restore the efficacy of anti-CTLA-4 in germ-free or antibiotic-treated mice [125]. Likewise, *Cordyceps sinensis* polysaccharides (CSP) usage in cyclophosphamide-treated mice affected T helper cells differentiation via upregulating TLR and NF-κB components, increasing SCFAs level, and modulating the composition and diversity of gut microbiota; thus, CSP was recommended as prebiotics to alleviate cyclophosphamide side effects [185].

Based on the difference observed in microbiome composition between immunotherapy-responder and non-responder patients in different types of malignancy (Table 4), multiple clinical trials are conducted to evaluate the efficacy of FMT from ICIs-responders to non-responders to overcome ICIs resistance [186]. Reconstitution of non-responder gut microbiota by fecal microbiota transplantation from responder patients was effective in reducing the ICIs resistance and enhancing the efficacy of immunotherapy approaches [187, 188].

According to the importance of bacterial metabolites in gut homeostasis and carcinogenesis, it could be considered a promising approach for cancer therapy. For instance, preventing polyamines production and uptake and applying short-chain fatty acids have therapeutic potential in cancer treatment [189–191]. Karpiński et al. reviewed the number of bacterial bioactive compounds with anticancer properties [192]. Despite several studies on carcinogenic and/or potentially anti-cancer metabolites, most were performed in vitro, and the safe therapeutic dose for clinical usage has not been determined [193]. The intricate interaction between metabolites and the immune system or tumor microenvironment is another limitation in its clinical use [191]. The therapeutic efficacy of metabolites (such as short-chain fatty acids) is dependent on a variety of parameters, including concentration and tissue context. Given The impact of context on optimal concentration, the time of intervention and metabolites delivery to the appropriate site should be considered. On the other hand, since some metabolites are carcinogenic, inhibition of their production process is helpful, but the investigation tools for their pharmacological inhibition are limited [194].

In comparison between several types of conventional anti-cancer treatment (like immunotherapy and chemotherapy) and microbial therapy, it should be noted that the former strategies have multiple disadvantages, including normal tissue toxicity, penetration to solid tumors, and drug resistance [195]. Cytotoxic chemotherapy is the main anti-cancer regime in patients with metastatic CRC [196]. As reported by Jessup and colleagues, the use of adjuvant chemotherapy in stage III colon cancer enhanced patients’ survival; however, its clinical benefit is lower in black persons or patients with high-grade cancer [197]. Immunotherapy, particularly Immune checkpoint inhibitors, is still in the beginning phases of gastrointestinal cancer treatment compared to melanoma and non-small-cell lung cancer (NSCLC). According to Ganesh et al. pembrolizumab and nivolumab, anti-PD1 antibodies, exhibited clinical advantages in patients with dMMR–MSI-H metastatic CRC (mismatch-repair-deficient or have high microsatellite instability). Contrarily, they have limited efficacy in the pMMR–MSI-L group, which compose the majority of patients with metastatic CRC. These types of CRC have Low immune cell infiltration and lower mutational burden. Combining ICIs with chemotherapy and radiotherapy may enhance their clinical efficacy by increasing T cell infiltration [198]. Despite advances in chemotherapy and immunotherapy in the survival of CRC patients, they have been associated with adverse effects such as colitis, diarrhea, and other immune-related adverse effects. Immune evasion and low mutational rates are also two hurdles in immunotherapy to treat gastrointestinal (GI) cancer [199]. Of note, evidence has demonstrated the association between the microbiome and the therapeutic efficacy of cancer treatments. Furthermore, microbiome-based therapies have fewer side effects than traditional treatments; however, more clinical research is necessary. Bacillus Calmette–Guerin (BCG) is the only microbial-based treatment approved by U.S. Food and Drug Administration (FDA). Given the inability of microbial therapy to entirely eliminate tumors, it is helpful to combine it with other conventional treatments. Additionally, bacteria delivery to tumor tissue should be optimized to minimize the possibility of systemic infections [195].

## Employing microbes or their metabolites as a predictable marker for clinical response or cancer progression

Immune checkpoint molecules were upregulated in several types of cancer to evade the anti-tumor immune response. So checkpoint inhibitors are applied to reinvigorate the anti-tumor immune response. Still, they could have also activated autoreactive T cells in various organs, such as the gastrointestinal tract, leading to immune-related colitis [200]. Determining the baseline composition of gut microbiota elucidated the association between *Firmicutes* enriched microbiota (e.g., *Faecalibacterium*) and clinical response or immune-related adverse events following ipilimumab treatment; thus, suggested as a marker for predicting clinical outcomes and colitis before ipilimumab therapy [201].

Furthermore, according to research conducted by Nomura et al. the concentration of fecal and plasma SCFAs is correlated with the effectiveness of anti-PD1 immunotherapy. The SCFAs concentration in responder and non-responder groups was higher in feces and plasma samples collected before anti-PD1 treatment (nivolumab or pembrolizumab). As a result, fecal SCFAs analysis was recommended as a non-invasive patient screening approach [202].

Given that bacterial genotoxins, including colibactin and *Bacteroides fragilis* toxin (BFT), play a role in colorectal cancer development, it may be beneficial to utilize as a marker for non-invasive screening of sporadic CRC in combination with a fecal occult blood test (FOBT) [203, 204].

Due to the association between microbiota-derived metabolites and CRC development, several reports highlighted the importance of metabolites as markers for CRC screening. For instance, stool metabolites analysis revealed greater levels of butyric acid and acetic acid in patients with colorectal adenomatous polyps (CAPs) (precancerous lesions of CRC) and higher t10,c12-CLA in healthy individuals [205]. As documented by Xi et al. microbial metabolite contents are varied throughout the different pathogenic sites of CRC [206]. Furthermore, Gas Chromatography–Mass Spectrometry (GC–MS) examination of fecal specimens from CRC patients and healthy people revealed higher concentrations of acetate and amino acids in CRC patients, as well as higher concentrations of butyrate and ursodeoxycholic acid in healthy individuals [207]. Another GC–MS-based metabolomics analysis discovered a higher level of polyamines and amino acids in CRC patients [208]. It's worth mentioning that the outcomes of some investigations were inconsistent. For example, there was no link between fecal SCFAs (acetate, butyrate, and propionate) concentration and tumor status in a study conducted by Sze and colleagues [209].

In addition to the predictive potential of microbial markers in the detection/prognosis of colorectal cancer, its usage is associated with limitations. (1) The complexity and diversity of microbiome between individuals and various populations complicated the detection of universal microbial markers. (2) Due to the variation of different studies in sample collection, metabolite and RNA extraction, and data analysis that impact the determination of microbial markers, standardization is essential to compare the outcomes of studies. (3) The presence of unrelated microbial species in the stool sample makes it difficult to detect biomarkers of the disease. (4) It is pertinent to point out that antibiotics usage alters the microbial community and the expression of markers. (5) The differences between mucosa-associated and fecal microbiota should be considered [210, 211].

## Conclusion

**Fig. 2:**
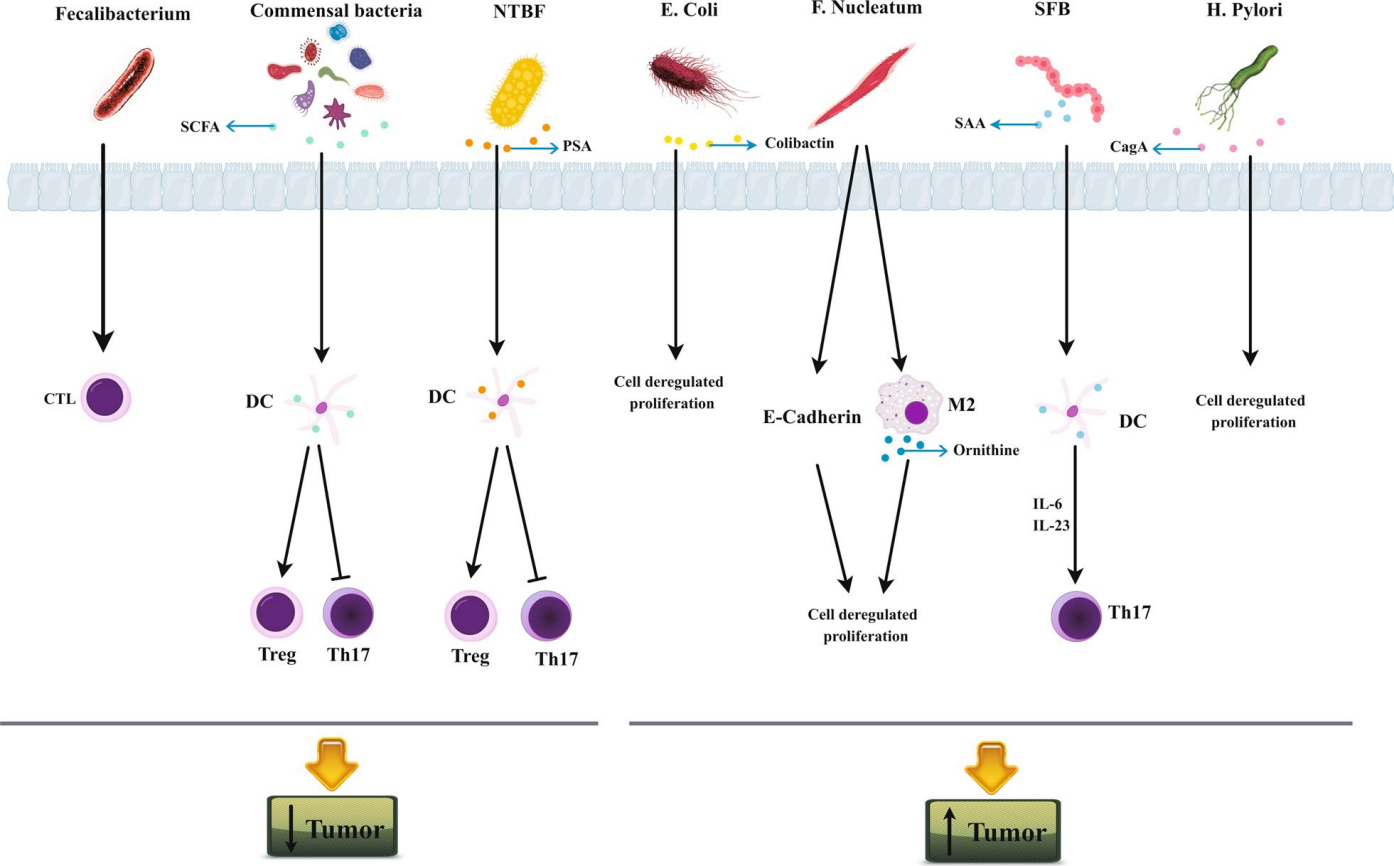
The underlying mechanisms behind the role of gut microbiota in preventing or facilitating tumor progress. Non-toxigenic Bacteroides fragilis (NTBF), Polysaccharide A (PSA), Short-chain fatty acids (SCFA), Serum amyloid A (SAA), Segmented filamentous bacteria (SFB), Cytotoxin-associated gene A (CagA)

Microorganisms (including bacteria, fungi, viruses, and protozoa) are inhabited on all body surfaces and affect various aspects of host physiology, such as metabolism and immune system development. They might act dual role in tumor treatment or progress by various mechanisms. In particular, they could inhibit or support tumor progress by deterring or promoting pro-tumor inflammation, respectively (Fig. 2).

Dysbiosis, or the alteration of the gut microbial community, is caused by environmental or host-related changes and influences the incidence and progression of diseases such as cancer. Indiscriminate antibiotics usage potentiated cancer initiation and progression by depleting health-promoting bacteria, promoting pathogen colonization, and reducing microbial diversity. Hence, restoring normal flora by taking probiotics supplements or other dietary interventions is beneficial for maintaining optimal microbiota composition. In addition, more pathogen-specific antibiotics with a narrow range and developing novel anti-bacterial strategies (like nano-medicine) aid in preserving eubiosis [183, 184].

The interaction between gastrointestinal microbiota and cancer progression is bidirectional, implying that tumor advancement impacts tumor/gut microbiota and vice versa. As a result, irrespective of whether the gut dysbiosis is the consequence of malignancy or the cause, it would be worthwhile to investigate the microbial community as a predictor of disease progression and response to treatment. Furthermore, since microflora may play a role in modifying the curative efficacy of anti-cancer therapy and alleviating their adverse effects, any changes in their diversity or population can affect the clinical outcomes of anti-cancer approaches. This study recapitulated the information that confirmed the link between gut microbiota and cancer. Despite extensive research in this area, only a few prospective studies have documented the causative role of microbiota in tumor initiation. Hence, further research is required to determine the mechanisms of microbial involvement in cancer occurrence to prevent carcinogenesis.

Despite huge advances in cancer treatment, not all individuals respond to treatment in the same way. At least in part, these discrepancies may be linked to microbial population diversity. Accordingly, identifying the bacteria involved and modulating the microbiome through dietary interventions, FMT, and antibiotics administration can improve response to therapy. Cancer bacteriotherapy (use of bacteria in cancer treatment, either alone or in combination with other therapies) can be utilized for the targeted delivery of therapeutic agents. Although the use of bacteria in cancer treatment has long been discussed, it has recently received more attention as a novel anti-cancer approach.

## Data Availability

Not applicable.
